# National and Sub-National Delivery of Balanced Energy and Protein (BEP) Supplements to Pregnant and Lactating Women in LMICs: Lessons from Multi-Country Implementation Case Studies

**DOI:** 10.3390/nu18091471

**Published:** 2026-05-05

**Authors:** Mihaela C. Kissell, Kaosar Afsana, Sufia Askari, Rimu Byadya, Ranadip Chowdhury, Parul Christian, Saskia de Pee, Lieven Huybregts, Fyezah Jehan, Tsering P. Lama, Anne C. Lee, Elisabeth T. Mukendi, Nafissa Osman, Isabel Potani, Lisa Rogers, Vani Sethi, Martin N. Mwangi

**Affiliations:** 1 Healthy Mothers Healthy Babies Initiative, Micronutrient Forum, Washington, DC 20005, USA; elisabeth.mukendi@micronutrientforum.org (E.T.M.);; 2 James P. Grant School of Public Health, BRAC University, Dhaka 1212, Bangladesh; 3 Sight and Life Foundation, 4303 Kaiseraugst, Switzerland; 4 Nutrition and Food Safety, World Health Organization, 1211 Geneva, Switzerland; 5 Society for Applied Studies, New Delhi 110016, India; 6 International Health and Human Nutrition, Johns Hopkins Bloomberg School of Public Health, Baltimore, MD 21205, USA; 7 Nutrition Division, World Food Programme, 00148 Rome, Italy; 8 Nutrition, Diets, and Health Unit, International Food Policy Research Institute, Washington, DC 20005, USA; 9 Department of Paediatrics and Child Health, Aga Khan University, Karachi 74800, Pakistan; 10 Nepal Nutrition Intervention Project Sarlahi, Kathmandu 355, Nepal; 11 Department of Paediatrics, Warren Alpert Medical School, Brown University, Providence, RI 02912, USA; 12 Department of Obstetrics and Gynochology, Eduardo Mondlane University, Maputo 1100, Mozambique; 13 Global Health Research Department, Ripple Global Health, London W1W 5PF, UK; 14 Global Practice for Child Nutrition and Development—Center of Excellence, UNICEF, Bangkok 10200, Thailand; 15 Division of Human Nutrition and Health, Wageningen University & Research, 6700 Wageningen, The Netherlands

**Keywords:** balanced energy and protein, implementation, pregnancy, antenatal care, maternal nutrition, supplementation, LMICs

## Abstract

The World Health Organization recommends the use of balanced energy protein (BEP) supplements during pregnancy in settings with a ≥ 20% prevalence of underweight women of reproductive age to reduce the risk of adverse health outcomes. Several countries are implementing BEP supplementation in varied formats. However, the implementation and monitoring of outcomes remain poor across countries. This qualitative study explores the experiences, opportunities, and challenges related to implementing national and sub-national BEP supplementation programs in nine countries (12 countries originally invited) to inform best practices. Methods: Semi-structured interviews were conducted with 15 personnel involved in its implementation in Haiti, India, Malawi, Mexico, Nigeria, Pakistan, Rwanda, Senegal, and Sri Lanka between October 2024 and March 2025. The interviewees in each country were predominantly implementation experts but also government officials involved in the provision of BEP supplementation. The transcripts were analyzed thematically, focusing on acceptability, adoption, appropriateness, cost, feasibility, and sustainability of outcomes. Results: In non-humanitarian settings (five countries), BEP supplementation was commonly integrated into the governmental health system or social protection programs. However, humanitarian contexts (four countries) often relied on partner-led (e.g., UN organizations) implementation. Clear operational protocols, including behavioral change communication strategies, facilitated the implementation. Community-based organization partnerships strengthened adherence; however, implementation costs, stock shortages, and geographic inequities in coverage varied and were limiting factors in scale-up, primarily in humanitarian contexts. Conclusion: In sum, two distinct implementation pathways emerged: government-led models characterized by policy integration, national ownership, and more stable systems, and humanitarian or donor-led models shaped by crisis response, external dependency, and non-committal challenges. Successful implementation of BEP supplements depends on the presence of effective policies, context-adapted design, integration into health systems, consistent funding, and effective monitoring. There is a need for implementation research to generate evidence on best practices when implementing BEP supplementation programs.

## 1. Introduction

Maternal undernutrition is prevalent globally, particularly in low- and middle-income countries (LMICs) [1]. Various indices have been used to estimate maternal undernutrition, including short stature (<150 cm; affecting approximately 450 million) [2], underweight (Body Mass Index (BMI) < 18.5 kg/m^2^; affecting approximately 240 million), and anemia (affecting approximately 468 million) [3]. Several adverse maternal and child health outcomes have been strongly linked with maternal undernutrition, e.g., small for gestational age or low birthweight (<2500 g), which affects 20.5 million births globally [4]. Maternal and child nutrition interventions are critical in combating undernutrition and decreasing health disparities in food-insecure settings [5]. Thus, the World Health Organization (WHO) recommends balanced energy and protein (BEP) supplementation at population level to pregnant women within routine antenatal care (ANC) (e.g., hospitals, primary healthcare facilities, community locations, homes, etc.) in undernourished populations [6]. WHO defines undernourished populations as having ≥20% of women of reproductive age identified as undernourished, using a BMI < 18.5 kg/m^2^ or a low mid-upper arm circumference (MUAC; no cutoff specified) [6].

Multiple formulations of BEP supplements have been used in research studies and programs. A narrative review of systematic reviews and meta-analyses [7] points out that when BEP supplements are compared with control (typically iron and folic acid (IFA) supplements), they can improve birthweight and reduce the risk of stillbirth and being small for gestational age. However, there was variability in the types and nutritional composition of BEP supplements used [7]. For example, the energy content ranged from 118 to 1017 kcal, protein from 3 to 50 g, and fat from 6 to 57 g. To address this variability, a 2017 Expert Consultation Report, led by the Gates Foundation, provided recommendations for the macro- and micronutrient composition of BEP supplements [8].

Although WHO recommends this intervention, only a few countries (e.g., Mexico, India, Sri Lanka, Rwanda, and Pakistan) have implemented this intervention nationwide through domestic financing. Most BEP supplements are delivered in humanitarian settings in partnership with the World Food Programme (WFP) and other non-governmental organizations (*n* = 29 countries) [9]. In humanitarian settings, BEP supplements are typically delivered outside of ANC, with personnel reporting a lack of knowledge regarding the definition and composition of the supplement, its beneficiaries, and how it should be administered in conjunction with other WHO-recommended interventions within routine ANC [10]. There is, therefore, a critical evidence gap regarding the various aspects of implementing BEP supplementation at the population level for undernourished populations and at the individual level for women who are underweight for management/prevention of undernutrition in pregnancy and postnatally in real-life settings.

In response, we aimed to conduct detailed case studies in countries where BEP supplementation has been implemented at the national or sub-national level, in development settings or emergency/humanitarian settings. The goal was to elucidate the types of supplements used and to identify processes, challenges, opportunities, and experiences related to their use, potential targeting strategies, associated costs, delivery mechanisms, and training materials for health staff.

## 2. Materials and Methods

### 2.1. Study Design

We conducted case studies employing qualitative research methods.

### 2.2. Qualitative Data Collection Methods

We explored opinions and experiences of personnel involved in the implementation of BEP supplementation. Based on a prior literature review, we formulated open-ended interview questions (Appendix A), which were used to conduct in-depth interviews (IDIs) or focused group discussions (from October 2024 to March 2025), depending on the participant’s preference. The interviews were recorded (with participant approval) using a remote platform, such as Microsoft Teams (version 26072.521.4595.7966), and the data were transcribed. All participant identifier data were anonymized from the point of recording to maintain confidentiality.

### 2.3. Country Identification and Participant Selection

Through a desk review, we identified countries providing BEP supplementation at the national or sub-national level through government and/or non-government support [9]. We sought to include countries primarily from South Asia and sub-Saharan Africa, as these regions have the highest burden of women at risk of food insecurity and undernutrition [11,12]. We invited participants from 12 countries, and participants from nine countries responded positively to our request. The participants who agreed to participate (*n* = 15) were national/sub-national-level government officials or non-government officials involved in the implementation of BEP supplementation. They were identified through the desk review and referrals from professional contacts within the investigators’ network. We conducted qualitative IDIs with representatives in seven countries, Haiti, Mexico, Nigeria, Pakistan, Rwanda, Senegal, and Sri Lanka, and focused group discussions in Malawi and India with government and non-government officials and/or implementation experts to examine the implementation of BEP supplementation programs for pregnant women and, in some contexts, lactating women. Of note, in this paper, the term ‘pregnant and lactating women (PLW)’ is used in line with global nutrition program and product nomenclature (e.g., LNS-PLW) and refers to women during pregnancy and postpartum period when breastmilk production may occur.

### 2.4. Definition of BEP Supplements

For the purposes of this study, BEP supplementation is defined as the provision of food or nutritional products (e.g., locally available nutrient-dense foods or snacks, fortified cereals or flours/fortified blended cereal/flour, ready-to-use products such as lipid-based nutrient supplements) designed to provide a balanced intake of energy and protein (up to 25% of total energy from protein), as typically defined in prior evidence [13] aimed at improving maternal nutrition during pregnancy.

### 2.5. Data Analysis

We conducted interviews predominantly in English but also in French and Spanish for participants who were not proficient in English. Then, French and Spanish transcripts were translated into English. All English transcripts were uploaded, managed, and coded using Dedoose software (version 9.2.22). Initially, we developed a codebook based on the topics that the interview questions are organized under (Appendix A); for example, the code name was “Targeting” for the information captured under Topic #2: “Targeting strategies used.” Two researchers independently analyzed the transcript data for themes and subthemes using a combination of thematic analysis methods. Thematic analysis of data from in-depth interviews identified several key themes and sub-themes related to the implementation of BEP supplementation programs. These themes were organized using the implementation framework proposed by Procter et al., which encompasses the core domains of acceptability, adoption, appropriateness, costs, and feasibility; we were limited in time and resources to explore the rest of the implementation domains [14]. The findings were then summarized by theme. To understand the nutritional composition of various BEP formulations used, we conducted desk research and tabulated the findings.

### 2.6. Ethics and Confidentiality

This study did not require ethical approval as consent was requested and gained from all interviewees based on the following policy (46.104 section 2): https://www.ecfr.gov/current/title-45/subtitle-A/subchapter-A/part-46/subpart-A/section-46.1 (accessed on 7 March 2026). All data were stored securely and accessed only by the research team. The findings are reported in a manner that prevents the identification of any individual, ensuring the confidentiality of responses throughout the research process.

## 3. Results

A total of 15 experts participated in the study. Most (60%) participants were from the UN organizations; however, in their roles, they supported government programs. Other (40%) informants worked within the respective country’s Ministry of Health department. Given the small sample size, the breakdown of participants per country is not provided to avoid the potential issue of de-identifying participants. A thematic analysis of data from IDIs identified several key themes related to the implementation of BEP supplementation programs. These themes were structured using an adaptation implementation framework and included BEP supplement policy adoption; BEP supplement sourcing and production channels; materials and human resources used; BEP supplement cost, storage, and delivery; BEP supplement selection and acceptability, screening procedures; and BEP supplement duration, intake adherence, and sharing behaviors.

### 3.1. BEP Supplementation Policy Adoption

Based on IDIs, countries adopted BEP supplementation to address persistent maternal undernutrition and integrated it into pre-existing earliest point of contact delivery platforms (national health/social protection systems or humanitarian programs). Countries with long-standing programs delivering BEP supplementation and those that initiated their program independently based on the country situation (prior to the release of the WHO 2016 ANC guideline [6]) include Rwanda, India, Sri Lanka, Nigeria, and Mexico (Table 1). For example, India integrated BEP supplementation into the Integrated Child Development Services (ICDS) as hot-cooked meals or take-home rations; Sri Lanka incorporated Thriposha into a welfare-oriented health system with near-universal ANC coverage [15].

“*FBF [Forftified Blended Food, Shisha Kibondo] was chosen, but it has been a flagship programme for more than a decade in the country where there is a local production [by an] African group food [Africa Improved Foods company].*”

(Rwanda respondent).

Pakistan, in collaboration with UN agencies, currently implements a social protection program for PLW called “Benazir Nashonuma Programme” and provides a locally developed lipid-based nutrient supplement called “Maamta.” In Malawi, Haiti, Nigeria, and Senegal, implementing an often imported BEP supplement in the humanitarian or partner-led contexts linked it in response to emergency nutrition or food insecurity.

“*It is in this sense that each year, we try to implement a targeted program to target these vulnerable groups who live in departments identified as in crisis or in emergency, according to the results of an analysis called the Harmonized Framework, so based on this, we provide our assistance according to the modalities that are operationally feasible*”

(Senegal respondent)

National platforms adopted a blanket/universal approach within program eligibility, whereas humanitarian programs prioritized geographies in crisis and highly vulnerable households.

### 3.2. BEP Supplement Selection and Acceptability

Across the nine countries, the type of BEP supplement was primarily influenced by local habits, food practices, and infrastructure constraints, including access to water, fuel, and storage. The two main formulations used were a corn–soya blend fortified with essential micronutrients (CSB+) and lipid-based nutrient supplements for pregnant and lactating women (LNS-PLW), with preferences varying across cultures and contexts. For instance, in Nigeria, both formulations were provided; however, although IDIs reported that women liked LNS-PLW, the CSB+ form was a better fit because it could be prepared in various ways according to local cultural practices. In places without access to clean water, such as areas of Haiti, LNS-PLW was considered suitable because it does not require cooking. Furthermore, the IDIs shared that aside from women, the broader community also expressed favorable views towards the BEP products.

“*Much more preferred in my opinion should be the Super Cereal Plus [CSB++] because it aligns with the pattern, our consumption pattern, food preparatory pattern of women in the communities*”

(Nigeria respondent)

“*They love CSB and not only pregnant women, even their husbands too, love it because it’s delicious … When you say CSB they know what it is, and they like it, and … it’s them who gave me recipes, I collected their recipes and what they are used to preparing and because they think that it’s not just porridge that they can do, they can do other things with it.*”

(Haiti respondent)

Several countries also emphasized the role of locally developed and nutritious food baskets (no additional details provided) as alternatives to imported BEP products. In Senegal, for example, the government and its partners experimented with nutritious food baskets that included locally available foods for purchase at a modest price of USD 0.18–0.36 (~200 g sachet) and providing nutrition education. This approach aimed to enhance sustainability and cultural alignment while maintaining nutritional adequacy. Similarly, Haiti’s Akamil, a locally developed fortified flour blend originally designed to treat child malnutrition, was adapted for use by pregnant women, reflecting national innovation in the formulation of BEP supplements. Sri Lanka’s Triposha, Pakisan’s Maamta, Rwanda’s Shisha-Kibonbo, and Mexico’s Nutrivida were locally produced, and India provided hot cooked meals or take-home rations through Anganwadi centers, prepared using standard recipes and easily integrated into familiar local dishes. Overall, the participants said that product acceptability was high across all settings, largely because women could adapt BEP supplements to familiar recipes or because they valued the perceived health benefits for themselves and their babies.

Among the BEP products reviewed, LNS-based supplements of medium-/large-quantity and fortified blended flours (e.g., CSB type) were the only type of products that fully met the Expert Consultation Report’s definition of BEP supplementation (Table 2), providing the recommended 250–500 kcal/day or double this amount in high-risk areas, balanced protein (<25% of energy), and a comprehensive micronutrient profile [8]. Aside from India’s hot cooked meals/take-home rations, all BEP products were fortified with micronutrients.

### 3.3. BEP Supplement Sourcing and Production Channels

Across all nine countries, two major BEP supplementation sourcing models emerged: locally produced BEP supplements integrated into national health programs (typically government funded) and internationally sourced BEP supplements procured through partnerships with global UN agencies such as WFP and UNICEF and often distributed through national health platforms.

As shown in Table 1, countries with established local manufacturing capacity, including Rwanda, Sri Lanka, India, Pakistan, and Mexico, rely on domestically produced nutrient-dense ingredients. For example, in Rwanda, the government partnered with Africa Improved Foods, a local company certified by WFP, to produce Shisha-Kibondo, a maize–corn blend fortified with essential micronutrients (similar to CSB+) and distributed nationwide through health and social protection systems. Similarly, Sri Lanka’s BEP supplement, Thriposha (pre-cooked, fortified cereal-based flour) was given to all PLW. The ICDS program in India relied on both public and privately contracted mills to produce fortified blended foods and take-home rations, which are distributed through community centers, and to prepare hot cooked meals in community feeding centers. Further Indian PLW can obtain USD 70–75 in three installments if they attend the community feeding center or health facilities within the program, Pradhan Mantri Matru Vandana Yojana y. Pakistan, in collaboration with WFP, provides a BEP supplement in the form of LNS-PLW called Maamta, which is produced locally by Ismail Industries (WFP-certified). The Maamta sachets are distributed through the Benazir Nashonuma Programme that targets the poorest of the poor households. In Mexico, a fortified milk powder called Nutrivida was produced domestically by Liconsa, a semi-public enterprise under government oversight, and distributed through the Oportunidades conditional cash transfer program. In contrast, countries without large-scale production capacity, such as Haiti, Malawi, Nigeria, and Senegal, relied heavily on donor-driven procurement as highlighted by the Haiti representative “*It’s all on the partners*”. In these settings, WFP and UNICEF were responsible for sourcing and supporting the distribution of certified products, primarily CSB+ and LNS-PLW. In Haiti, WFP procured and imported CSB+ and distributed it in collaboration with national authorities and non-governmental organizations, alongside milk powder. Senegal’s UN organizations sourced a mix of fortified blended cereal/flours and LNS-PLW through multiple donor-supported mechanisms under the National Protocol for the Management of Malnutrition.

### 3.4. Materials and Resources Used in Program Implementation

Beyond product sourcing, BEP supplementation required a robust set of educational, operational, and monitoring materials to ensure safe use, proper preparation, and consistent distribution (Table 3). These operational tools were developed by governments, UN agencies, and NGO partners, and helped raise beneficiary awareness and ensure adherence.

Training and educational materials were widely utilized to enhance knowledge among both healthcare workers and beneficiaries. In India, the ICDS program provided standard recipes for hot cooked meals and take-home rations, supplemented by government-issued guidelines on food storage, hygiene, and safety. Wall posters and pamphlets translated into local languages were used to promote consistent food preparation practices.

“*There are some letters and guidelines available. For example, … there are some letters available from the government, for example, storage of such food, food handling, ensuring that the food is safe.*”

(India respondent)

During its operational phase, Mexico’s program developed training manuals and posters for health providers illustrating the nutritional value, preparation, and storage of fortified milk powder. Rwanda combined BEP supplementation with social and behavioral change communication (SBCC) materials, including counseling cards, peer-to-peer discussions, and cooking demonstration tools to promote maternal nutrition education. Pakistan distributed each LNS-PLW sachet detailing dosage (one sachet daily (75 g) for prevention and two (150 g) for treatment), storage conditions, and the importance of not sharing the product with family members.

“*So, it’s two sachets per day for a treatment. So, for stunting [prevention of undernutrition] it’s the same product, but like the dosage is low in preventive, we only give one sachet per day*”

(Pakistan respondent)

Haiti developed recipe cards and illustrated posters in Creole and French, informed by focus group discussions with women who shared local cooking practices. Similarly, Senegal utilized protocol-based training materials that provided practical instructions on hygiene, rationing, and the duration of consumption.

### 3.5. Human Resources Supporting the BEP Supplementation Implementation

Health professionals formed the first line of delivery of BEP supplementation in most countries (Table 1). The distribution of BEP supplementation was often integrated within ANC services and supervised by doctors, nurses, midwives, and at times nutrition officers. In Mexico, Rwanda (community health workers referred PLW to ANCs), Sri Lanka, and Pakistan, health professionals were responsible for screening pregnant women, providing BEP supplements, and offering individualized health and nutrition counseling. Public health midwives in Sri Lanka played a dual role in the distribution and health education, while nutrition managers and program officers at national and district levels oversaw program coordination and data reporting.

“*Some of them are paramedics, some of them are nurses, some of them are just social workers providing social mobilization or social behavioral change communication kind of activities or some support.*”

(Pakistan respondent)

At the community level, frontline workers and volunteers extended program reach. For example, India’s community workers called Anganwadi workers, supported by helpers, prepared and distributed meals or take-home rations, recorded attendance, and maintained household nutrition records. In Malawi, care groups and community volunteers are used to facilitate nutrition counseling sessions, organize meetings with local leaders, and reinforce key messages on product use. Community health workers also supported the implementation of BEP supplementation in Haiti, Nigeria, and Senegal, distributing the supplement in community centers or sometimes conducting home visits, distributing supplements, and providing hygiene education. Pakistan’s Community Management of Acute Malnutrition (CMAM) program engaged paramedics, social mobilizers, and contractual staff, who were funded by the Benazir Income Support Programme. Meanwhile, in Senegal, community actors (not necessarily qualified personnel) and women’s groups provided health education and follow-up support on BEP supplementation at the local level.

In countries where the intervention was delivered only in emergency or humanitarian settings by a non-governmental organization (NGO), it was often provided by individuals with or without a formal healthcare background. These included trained community health workers or volunteer health staff (e.g., Doctors Without Borders), behavioral change communication staff, or other volunteers (unpaid) at the community level, who provided group counseling (offering multiple services beyond BEP supplementation) and individual counseling, as seen in the cases of Nigeria, Senegal, and Haiti.

“*Programme starts from the non-governmental organization, also supported by community-based staff like community volunteers that have been trained on how to distribute these commodities. But really nothing much technical around it.*”

(Nigeria respondent)

### 3.6. BEP Supplement Cost, Storage, and Delivery

Respondents were not aware of the costs of the BEP supplements and mentioned that the information should be publicly available in government reports or in the case of imported products, respondents guided us to check the UNICEF Supply Catalog or contact WFP officers. Nonetheless, the BEP supplement provided was free of cost for the PLW recipients.

Geographic targeting for BEP supplementation varied across countries, reflecting differences in program maturity, vulnerability mapping, and available funding. In countries where BEP supplementation was integrated into the health system, three main delivery platforms were identified: health facilities integrated with ANC services, community-based locations (e.g., health posts), and nutrition treatment programs in facility or community, such as CMAM (Table 1).

National social protection programs in Mexico, Pakistan, Rwanda, and Sri Lanka relied primarily on ANC-linked health facilities to ensure coverage and continuity. In Pakistan, BEP supplements were distributed through CMAM units adjacent to public hospitals (to obtain proof of pregnancy and trimester), with pregnant women attending monthly for four months of supplementation. India, Mexico, Rwanda, Pakistan, and Sri Lanka provided national-scale BEP supplementation, typically integrated into social protection systems and delivered through government-managed programs. In India, centers were established in every village under the ICDS, while Mexico’s former Oportunidades program, Rwanda’s national maternal nutrition platform, and Sri Lanka’s Thriposha initiative each embedded BEP supplementation within their respective health or welfare systems.

“*The center is equipped with two female workers. One is the Anganwadi worker, who’s the leader, main female worker and she has a support staff like an Anganwadi helper, the center helper… these two females manage the center and ensure the distribution of the cooked food as well as the take [home] package.*”

(India respondent)

In countries with sub-national programs, approaches were tailored to the local context. In Haiti, the implementation was largely donor-driven and geographically limited, as there was no national BEP supplementation program to support all PLW. The distribution depended on partner presence and local familiarity, with WFP and collaborating NGOs operating platforms in selected regions. In Malawi, the implementation of BEP supplementation was planned for concentration in two southern districts identified through surveys as having a high prevalence of maternal undernutrition (MUAC < 21 cm) and severe food insecurity following El Niño weather-related shocks. Similarly, Senegal targeted regions experiencing crises based on the Harmonized Framework Analysis, while Nigeria used a five-phase Integrated Food Security Phase Classification tool to identify high-risk zones (phases 3–5) for BEP supplementation rollout for all PLW. When resources were constrained, BEP supplementation was prioritized during the lean or preharvest season with distribution often in a central location within the community so women can pick up the supplements (at times, community workers also distribute supplements directly to PLW).

“*So, in the emergency contexts, these [BEP supplements] are delivered through the health system. So, when they [PLW] come to the health facility… but usually because it’s implemented as a form of food distribution, sometimes it’s distributed at the health facility, sometimes distributed at the community level.*”

(Nigeria respondent)

In Pakistan, Haiti, Malawi, Nigeria, and Senegal, partner organizations such as WFP, UNICEF, and local NGOs were integral to BEP supplementation implementation, often maintaining temperature-controlled warehouses and handling stock rotation. WFP and UNICEF provided technical guidance and coordinated procurement, particularly in Pakistan, Rwanda, and Malawi, to ensure consistency with global standards. Local NGOs in India, Mexico, and Haiti conducted training sessions, awareness campaigns, and coordinated efforts between communities and local government offices.

There were challenges with storage and supply chain. For instance, in Sri Lanka, challenges included incomplete stock records, inconsistent monitoring of expiration dates, and inadequate ventilation or temperature control in district warehouses. Mexico utilized a paper-based warehouse logbook and transport checklist system to track stock movement between central warehouses and health facilities, though mismatched inventory levels were sometimes reported. In Pakistan, tracking registers and return logs were introduced to monitor sachet distribution and prevent misuse. Malawi plans to use a combination of counseling cards and local radio messages to support community-level monitoring and awareness. In Haiti, civil unrest complicated the supply chain of the BEP supplements.

“*The CSB, they were there, and we couldn’t get them out because the gangs, they’re in the hands of the gangs and they’re controlled by the armed groups and so we couldn’t get them out.*”

(Haiti respondent)

### 3.7. Screening Procedures, Criteria, Duration, and Challenges of BEP Supplementation

Across all countries, BEP supplementation targeted predominantly PLW using similar eligibility criteria, primarily based on nutritional status (MUAC), pregnancy confirmation, and vulnerability to food insecurity. Most national programs (India, Sri Lanka, and Mexico, when active) provide blanket BEP supplementation to all PLW enrolled in social protection or ANC platforms. In contrast, humanitarian and targeted nutrition programs (Malawi, Haiti, Nigeria, Senegal, Rwanda, and Pakistan) typically restricted eligibility to women with MUAC < 21 cm, women living in severely food-insecure households, or those registered in poverty-targeted social assistance programs. Some countries, such as Pakistan and Rwanda, use a two-tiered approach, offering a standard BEP supplementation dose for prevention to all eligible PLW in social protection programs, while providing higher or therapeutic doses to women who screen as malnourished. Screening was primarily conducted by health facility staff, community health workers, or program volunteers, depending on the delivery platform and resources available (Table 1).

In various countries, programs standardized the daily BEP supplement intake according to product type and local guidelines. For example, in Pakistan, pregnant women received one or more 75-g LNS-PLW sachets as a daily ration. Most countries reported that the duration of BEP supplementation was maintained throughout pregnancy, although some indicated that it continued for up to six months postpartum (Table 1). One participant quoted:

“*But like the benefit, the pregnant and lactating woman when they come to the health facility, they’re being screened, and if they’re diagnosed as having acute malnutrition, which we screen through the MUAC tapes, mid-upper arm circumference, and if their MUAC is less than 21.*”

(Pakistan respondent)

Across countries, BEP supplementation exit criteria fell into two main categories: those with clear, measurable discharge standards and those using time-based, non-clinical/nutritional exit points (Table 1). Countries with measurable standards mostly operated targeted or humanitarian programs, as follows. Pakistan discharged malnourished women once their MUAC reached ≥21 cm, while prevention beneficiaries exited at six months postpartum. Senegal applied objective recovery thresholds; women exited when MUAC reached ≥23 cm for treatment or after 3–6 months for prevention. Nigeria and Haiti followed emergency protocols, in which supplementation ended after 3–6 months or at 6 months postpartum, depending on the programme modality. Rwanda, although MUAC is used for entry (MUAC < 21 cm), applies a defined recovery-based exit criterion: women exit once they have achieved “sufficient nutritional recovery” as assessed through routine MUAC and weight-gain monitoring at ANC, rather than a fixed postpartum duration.

Conversely, countries without measurable discharge criteria relied on simple time-bound approaches. For example, Malawi provided BEP supplementation from the first ANC visit until four weeks postpartum. At the same time, India and Sri Lanka exited all PLW at six months postpartum under universal social protection platforms. Mexico (during its active phase) allowed continuation through one year postpartum.

“*…So once the pregnancy is identified and at the start of the 2nd trimester of pregnancy, each woman is offered this Triposha supplement… And they are continued throughout pregnancy. In fact, they’re continued even beyond pregnancy for a period of six months after delivery to cover the postnatal and the breastfeeding period as well.*”

(Sri Lanka respondent)

Screening challenges for BEP supplementation programs targeting PLW varied across countries but shared common issues. For instance, in Haiti, there was a confusion among implementors about which MUAC cutoff to use, 21 or 23 cm. Multiple countries faced funding difficulties in providing the supplement to all eligible PLW. For instance, Malawi could not provide supplements to people outside the districts currently targeted for BEP supplementation. This was also a challenge in India, where many centers were absent in cities. In Sri Lanka, the challenge laid in blanket supplementation, and whether it would be more beneficial to target only PLW with underweight and normal-weight and exclude those who are with overweight or obesity. The systems in these countries did not yet allow for clear entry and exit points in the programs, and pregnant women often did not disclose their pregnancy until it showed, complicating personalized follow-up and effective management.

“*It is not that easy for a woman to disclose pregnancy in the first trimester, mainly because of cultural beliefs, among other.*”

(Malawi respondent)

### 3.8. BEP Supplementation Intake, Adherence, and Sharing Behaviors

Despite general acceptability, adherence to prescribed dosing was a recurring challenge, with widespread indications of product sharing within households. In nearly all settings, Pakistan, Haiti, Malawi, Nigeria, Mexico, and Sri Lanka, experts reported that pregnant women often shared their BEP supplement rations with husbands, children, or other relatives, either out of cultural obligation or food scarcity. This practice frequently undermined the intended nutritional benefit for the mother. In some instances, it was reported that women also shared the supplement with their animals, as follows:

“*But we saw the blend was not used the way it should have been by the beneficiaries, giving it to their animals*”

(Pakistan respondent).

To address this challenge, several countries employed behavioral and monitoring strategies. In Pakistan and Senegal, staff made follow-up visits and random checks. Counseling highlighted the benefits and risks of the BEP supplements for mothers. Rwanda and Haiti’s programs, which incorporated SBCC components, peer support, visual demonstrations, and community leader involvement, reported to be more effective in improving adherence. Moreover, adherence was generally higher with LNS-PLW products compared to CSB, likely because LNS-PLW was viewed as a personal supplement rather than household food.

## 4. Discussion

Despite WHO recommending BEP supplements for pregnant women in settings with ≥20% of women underweight [6], there is limited operational evidence on BEP supplementation implementation programs. This study describes the implementation of BEP supplementation for PLW in Haiti, India, Malawi, Mexico, Nigeria, Pakistan, Rwanda, Senegal, and Sri Lanka. The study also identified various enabling factors and challenges affecting the implementation of BEP supplements. A key and universal enabling factor for implementing the programs was the national/sub-national adoption and operationalization of BEP supplementation, driven by a shared objective of addressing persistent maternal undernutrition and, in some countries, coupling it with a conditional cash transfer. However, countries varied in how the intervention was positioned, sourced, and targeted within their health and social protection systems. Diverting BEP supplements from PLWs to other family members is identified as a challenge in most settings, despite efforts to discourage this misuse. Funding limitations for nutrition interventions such as BEP supplements was also a common concern for respondents.

Two key enabling factors were the adoption of BEP supplementation in policy documents and creating a locally acceptable product; these aspects were particularly evident in countries such as India, Mexico, Rwanda, Sri Lanka, and more recently Pakistan. These settings integrated BEP supplementation into a routine package for PLW more systematically into their national health, nutrition, or social welfare programs. These contexts highlight how institutional continuity, but also existing domestic supply mechanisms, enable sustained access and national ownership of BEP supplementation. This is consistent with the existing literature, which has reported that strong maternal health policies are associated with higher utilization of maternal health services, as well as improvements in maternal and child health indicators [28,29,30]. By contrast, countries such as Malawi, Haiti, Nigeria, and Senegal operating in humanitarian, crisis-prone, or donor-dependent environments introduced BEP supplementation largely through emergency nutrition and food security programs. These countries relied primarily on development partners for product procurement, distribution, and sometimes even protocol definition. Arguably, PLW living in humanitarian settings are at a higher risk of undernutrition and its negative consequences than those outside these settings [29,30]; thus, there is a need for more sustainable BEP supplementation implementation strategies, unlike what was observed.

Establishing sourcing pathways for BEP supplementation products was also a key priority. Some countries sourced BEP supplements from domestically produced local foods (Rwanda, Sri Lanka, India, Pakistan, and Mexico), while others sourced internationally supplied foods (Haiti, Malawi, Nigeria, and Senegal). This underscores the importance of countries seeking to implement the BEP supplementation for PLW to explore locally available nutritious foods, which could potentially strengthen national ownership and reduce reliance on imports [6]. There have also been concerns that some imported lipid-based supplements (e.g., Plumpy nut) for children that are highly similar to lipid-based BEP supplements for pregnant women (e.g., LNS-PLW) could potentially disrupt local diets [31]. However, this issue has been raised in the context of treating undernutrition in children aged 6–59 months and may not apply to PLW, who belong to an older age group. Furthermore, although some contexts relied on imported BEP products, only those deemed acceptable by locals were implemented with little to no replacement of the usual diet by these supplements as evidenced in some settings [32]. There remains, however, a critical need to evaluate long-term outcomes of various BEP supplements in terms of their impact on dietary patterns.

While BEP supplements were generally well accepted by PLW (based on this study’s participants perspective), adherence challenges were reported across nearly all countries. In extreme cases, BEP supplements were diverted to livestock. This evidence is consistent with other studies reporting sharing of BEP supplements [33,34]. In our case study, we reported that some countries such as Rwanda, Malawi, and Pakistan used behavior change communication and monitoring; however, there is limited evidence on the effectiveness of these strategies in reducing the sharing of supplements with family members. Furthermore, it has been argued that sharing food supplements within the household may indicate household food insecurity [35,36] and potential undernourishment of other family members. This aspect highlights the need for interventions that target the whole family to ensure that PLW are adequately nourished [37,38].

In all countries, the implementation of BEP supplements was supported by operational guidance, behavior change tools, and training materials. The integration of educational behavior change tools in maternal and child health programs has been generally associated with enhanced uptake and effectiveness of interventions [39]. However, despite the availability of operational tools, some countries, such as Haiti, faced challenges in implementing screening and eligibility criteria that were often inconsistent, leading to confusion about MUAC cutoffs. Furthermore, some countries, such as Sri Lanka, which have universal targeting, struggled to exclude women with overweight or obesity due to universal coverage norms. In many settings, late disclosure of pregnancy further complicated early initiation of supplementation. These challenges highlight the need for more explicit global and national guidance. Earlier studies have reported education as the most common strategy for changing the behaviors of those accessing maternal healthcare. At the same time, health training was the most common strategy in studies targeting the behaviors of those delivering maternal healthcare [39]. Therefore, countries seeking to adopt BEP supplementation should consider health training that address barriers to effective uptake and effectiveness, ensuring that maternal undernutrition is reduced through BEP supplementation programs despite the availability of some operational tools.

Adequate training and human resources are also essential for the success of BEP supplementation programs. ANC-linked distribution models were prevalent and relied on doctors, nurses, midwives, and nutrition officers for screening, dispensing supplements, and counseling. In some settings, community workers and volunteers were utilized in community feeding centers. In non-emergency settings, these community health workers were typically highly experienced and trained in basic maternal health and nutrition. However, in some settings, particularly emergency settings that rely on unpaid volunteers who may have limited training; other reported resource challenges included gaps in warehouse documentation, temperature control, and stock monitoring. Donor-dependent programs were particularly vulnerable to stockouts or geographic fragmentation due to funding fluctuations. Consistent with existing literature, some humanitarian settings have experienced supply blockages, such as sub-Saharan African communities, due to armed groups blocking the movement of products from warehouses [40]. Because the implementation of BEP supplementation relies on continuous access and consistent dosing, such bottlenecks pose major risks to the program’s success.

The main strength of this study is its global representation, encompassing cases from Latin America, Africa, and South Asia, as well as major regions affected by maternal undernutrition. Furthermore, including countries with long-standing BEP supplementation programs, newly implemented programs, and phased-out BEP supplementation programs in both humanitarian and non-humanitarian settings enhances the representativeness of the findings. The main limitation is that, by nature of this being a case study, the findings are largely descriptive and provide limited inference on which implementation strategies for BEP supplementation were the most effective. Furthermore, the respondents interviewed did not include PLW who are the direct beneficiaries of BEP supplements. This omission creates a significant knowledge gap, limiting our understanding of the program from both the implementers’ and beneficiaries’ perspectives. Lastly, we only inquired about the cost of BEP supplement and who pays for it, but we obtained limited but publicly available information.

## 5. Conclusions

In conclusion, these findings suggest that the implementation of BEP supplementation depends on adherence to established protocols, strong delivery platforms, clear eligibility criteria, and communication strategies that support meaningful behavior change. Ensuring equitable access, particularly in fragile or food-insecure settings, is vital to achieving effective coverage for PLW. The findings also underscore the need for operational research to refine implementation strategies across diverse contexts, including greater engagement with PLW to understand their experiences with BEP supplements. Overall, two distinct implementation pathways emerged: government-led models characterized by policy integration, national ownership, and more stable systems, and humanitarian or donor-led models shaped by crisis response, external dependency, and non-committal challenges. While both approaches have expanded access, their contrast highlights the importance of sustained national commitment, standardized guidance, and resilient supply chains to ensure consistent coverage and adherence. Strengthening locally driven implementation, even in crisis-affected settings, alongside improved behavior change approaches and more transparent eligibility processes, will be essential to achieving long-term, equitable, and effective BEP supplementation programs.

## Figures and Tables

**Table 1:** Summary of BEP supplementation implementation during pregnancy and postpartum.

Country	Setting & Partners	BEP Formulation	Eligibility & Targeting	Delivery Platform	Delivery Personnel	Certification & Producer	Supply Chain Management	Duration & Exit	Funding/Social Protection	Special Notes
Malawi	Sub-national; UNICEF, Malawi Red Cross, Farmers Union	LNS-PLW	MUAC < 21 cm; ANC starts at 4 wks postpartum	ANC facilities	Health workers, CHWs	Multiple producers; UNICEF certified	UNICEF & health system imports & distributes	Pregnancy-4 wks postpartum	Donor-funded/none	Expansion planned in 2025
Rwanda	National; Government + Africa Improved Foods	CSB+ (Shisha Kibondo)	MUAC < 21 cm; social protection beneficiaries	ANC facility	Midwives, nurses, health officers	Africa Improved Foods (WFP-certified)	Producer distributes nationally	Until adequate weight gain & lactating women	Government-funded; social protection	Program initiated in 2016 [16]; strong community SBCC model coupled with ANC
Pakistan	National; BISP + WFP/UNICEF	LNS (Maamta)	All BISP PLW; double dose if MUAC < 21 cm	Health facility (for ANC & CMAM services)	Contract staff, CMAM workers	Ismail Industries (WFP-certified)	WFP manages procurement to last mile	6 mo postpartum or MUAC ≥ 21 cm	Government-funded; social protection & conditional cash transfer to get BEP	Program initiated in 2020 [17]; integrated CMAM + ANC
India	National; ICDS, Government + NGOs	Hot cooked meals or take-home rations	All PLW in ICDS; blanket recommendation	Feeding (“Anganwadi”) centers	Anganwadi workers & helpers	State/private producers (not certified)	Government-managed decentralized supply	2nd trimester–6 mo postpartum	Government-funded; conditional cash transfer program PMMVI ($70–75) as of 2017	Program initiated in 1974 [18]; highly decentralized (state specific)
Sri Lanka	National; Government + Thriposha Ltd.	CSB (Thriposha)	All PLW until 6 mo postpartum	ANC clinics	Public health midwives & nurses	Thriposha Ltd. (not certified)	Government-managed	Pregnancy–6 mo postpartum	Government-funded; social protection; blanket supplementation	Program initiated in 1973 [15]; two 750 g packs/month
Mexico	National until 2014; NGOs now	Fortified milk powder (Nutrivida); later MMS	PLW in social assistance program	ANC clinics	Doctors, nurses	Lincosa (not certified)	National distribution (when active)	Pregnancy–1 yr postpartum	Government-funded; social protection & conditional cash transfer	Program initiated in 1997 [19,20]; discontinued 2014–2018
Haiti	Emergency; WFP, UNICEF, NGOs	CSB+, milk powder; cash transfers	PLW in food-insecure households based on survey	Community centers; home visits	CHWs, partner staff	Mainly international manufacturers; UNICEF/WFP-certified	WFP warehousing & delivery	Pregnancy–6 mo postpartum	Donor-funded/none	Cash transfer in urban areas
Nigeria	Emergency; WFP, USAID, DFID, etc.	LNS-PLW, CSB+, premixes	MUAC < 21 cm	ANC; community centers; home visits	Community volunteers; CHWs	Mainly international manufacturers; mostly UNICEF/WFP-certified	Donor-managed supply chain	Pregnancy-6 mo postpartum	Donor-funded/none	Implemented in 26 states
Senegal	Emergency; Gov’t + UNICEF/WFP, HKI, etc.	LNS-PLW, CSB, premixes	MUAC < 21 cm; Harmonized Framework Analysis	ANC; community centers; home visits	Community actors	Imported certified products	Donor + government jointly manage	3–6 mo (prevention); 6 mo (treatment)	Multi-donor/none	Acceptability + PDM conducted

ANC: antenatal care; BEP: balanced energy– and protein supplementation; BISP: Benazir Income Support Programme; CHW: community health worker; CMAM: Community-based Management of Acute Malnutrition; CSB: Corn–Soy Blend; CSB+: Corn–Soy Blend Plus (“Super Cereal”); DFID: Department for International Development (UK); HKI: Helen Keller International; ICDS: Integrated Child Development Services; LNS: lipid-based nutrient supplement; LNS-PLW: lipid-based nutrient supplement for pregnant and lactating women (medium-/large-quantity LNS); MMS: multiple micronutrient supplement; MUAC: mid-upper arm circumference; NGO: non-governmental organization; PLW: pregnant and lactating women; PDM: post-distribution monitoring; PMMVY: Pradhan Mantri Matru Vandana Yojana; SBCC: social and behavior change communication; UNICEF: United Nations Children’s Fund; USAID: United States Agency for International Development; WFP: World Food Programme.

**Table 2:** Nutritional composition of different types of BEP supplements.

BEP Product Type	Producer/Sourcing Organization	Energy	Protein	Fat	Micronutrient Profile	Countries
Fortified blended flour/cereal (CSB+/Super Cereal) [21]	WFP/UNICEF standard	≈380 kcal/100 g	≤14 g/100 g	≥6 g/100 g	Fortified vitamin–mineral premix	Haiti, Nigeria, Senegal; equivalent blends in Rwanda
Shisha Kibondo (fortified blended flour/cereal) [22]	Africa Improved Foods, Kigali, Rwanda	Not available	Not available	Not available	Fortified vitamin–mineral premix (slightly different from Super Cereal)	Rwanda
Thriposha (fortified blended flour/cereal) [23]	Sri Lanka Thriposha Limited, Ja-Ela, Sri Lanka	≈402 kcal/100 g	20 g/100 g	7.8 g/100 g	Iron, iodine, full premix	Sri Lanka
LNS-PLW [24]	(UNICEF/WFP)	510–590 kcal/100 g *	18.8–22.2 g/100 g *	26–39.3 g/100 g *	Full PLW micronutrient premix	Pakistan, Malawi, Nigeria, Senegal; also in some Haitian settings
Nutrivida fortified milk powder [25]	Liconsa, S.A. de C.V., Mexico City, Mexico	250 kcal/54 g	12–15 g/54 g	Varies	Iron, sodium, zinc, iodine, vitamins C, E, B2, B12, folate	Mexico
Full-cream milk powder	Akamil (local product; multiple manufacturers)	≈480–500 kcal/100 g	24–26 g/100 g	25–28 g/100 g	Ca-rich; vit A/D if fortified	Haiti
Hot meals/take-home rations [26,27]	ICDS India, Mumbai, India	~600 kcal/day	18–20 g/day	Varies	Micronutrients differ by THR product; up to 50% RDA fortification	India throughout universal entitlement under food security act

* Product ration is 75 g. BEP: balanced energy–and protein supplement, CSB+: Corn–Soy Blend Plus, LNS-PLW: lipid-based nutrient supplement for pregnant and lactating women, WFP: World Food Programme, UNICEF: United Nations Children’s Fund, PLW: pregnant and lactating women, ICDS: Integrated Child Development Services, RDA: recommended dietary allowance, Vit: vitamin, Ca: calcium.

**Table 3:** BEP supplementation training practices, materials used, and implementation.

Country *	Key Practices	Materials Used	Implementation
India	Take-home rations by government-certified agencies or hot cooked meals prepared in feeding (“Anganwadi”) centers by women self-help groups federation using raw ingredients as an enterprise model.	Standard recipes and government guidelines on food safety.	Meals prepared on-site; take-home rations used at distribution-only centers.
Mexico	Development of training materials during BEP program.	BEP products, posters, and manuals for health providers.	Distributed to health providers who educated beneficiaries during program activities.
Pakistan	Stunting prevention and CMAM for PLW.	BEP supplement sachets, instructions on dosage to avoid supplement sharing.	Daily sachet distribution, usage tracking, and education on proper use and storage.
Rwanda	National maternal nutrition and ANC guidelines.	BEP supplements, SBCC packets.	Supplements paired with peer discussions, counseling, and cooking demos.
Haiti	Awareness training and recipe adaptation.	BEP supplements, recipe cards, posters with images and preparation instructions.	Materials on preparation, storage, and preservation in Creole/French; focus groups to inform content; awareness campaigns.
Malawi	Health messages and community involvement.	BEP supplements, counselling cards, and local radio messages.	Local meetings with chiefs and local leaders.
Senegal	National malnutrition management protocol with support from development partners.	Government-recommended BEP products.	Community guidance on hygiene, rations, and consumption duration.

Abbreviations: ANC: antenatal care; BEP: balance energy and protein supplementation; CMAM: Community-based Management of Acute Malnutrition; SBCC: social and behavior change communication. * We did not obtain information on this topic from Nigeria and Sri Lanka.

## Data Availability

The datasets presented in this article are not readily available because the interview data are from government officials and UN implementors who could be easily de-identified in each country. Requests to access the datasets should be directed to the corresponding author.

## Abbreviations

The following abbreviations are used in this manuscript:

ANC	antenatal care
BEP supplementation	balanced energy and protein supplementation
CMAM	Community Management of Acute Malnutrition
CSB	corn–soya blend
ICDS	India integrated BEP supplementation into the Integrated Child Development Services
IDI	in-depth interviews
LNS-PLW	lipid-based nutrient supplement for pregnant and lactating women
MUAC	mid-upper arm circumference
NGO	non-governmental organization
PLW	pregnant and lactating women
WHO	World Health Organization

## Appendix A. List of Interview Questions

### Appendix A.1. BEP Questionnaire

The Global Balanced Energy–Protein Supplementation—Technical Advisory Group (BEP-TAG) (https://hmhb.micronutrientforum.org/bep-tag/ accessed on 1 September 2024), led by Dr. Mihaela C. Kissell and hosted by the Micronutrient Forum (within the Healthy Mothers Healthy Babies Consortium) in collaboration with the World Health Organization (WHO), aims to interview government officials, maternal and child health nutrition experts, and health staff from countries where balanced energy and protein (BEP) supplementation has been rolled out at the national and sub-national (e.g., emergency/humanitarian) level to understand the BEP supplementation type, form, quantity, targeting strategies used, cost and supply, delivery mechanisms, monitoring process, and training materials for health staff. The information captured will inform the WHO implementation guidance and support optimizing BEP supplement interventions to improve maternal and child health outcomes in undernourished populations.

Instructions for the interviewer:

1. Inform/remind the interviewee that the interview is recorded and confidential.
2. Introduce yourself and explain the purpose of the interview, referencing the Healthy Mothers Healthy Babies Consortium and its goal to enhance BEP supplement interventions.
3. Inform the interviewee that we are trying to learn from their experience. The interview is not a professional or academic assessment of their knowledge of food supplementation/nutrition or antenatal care.
  a. Reassure throughout the interview as necessary that there are no wrong answers.
4. Ensure that the interviewee understands the definition of BEP supplementation (read the definition below).
5. Ask questions as they are presented in the form, allowing the interviewee to elaborate as needed. Probing further where necessary (e.g., understanding local nuances or specific challenges) is encouraged.
6. Maintain neutrality and avoid leading questions, ensuring that responses reflect the interviewee’s genuine experiences and insights.
7. Ensure clarity around each topic area (e.g., BEP supplement type, targeting strategies, monitoring process) before moving on to the next.

### Appendix A.2. Definition of BEP Supplementation

For the purposes of this study, based on prior evidence, BEP supplementation is defined as the provision of food or nutritional products (e.g., locally available nutrient-dense foods or snacks, fortified cereals or flours, and ready-to-use products) designed to provide a balanced intake of energy and protein (up to 25% of energy comes from protein, as typically defined in prior evidence [13]) that is aimed at improving maternal nutrition during pregnancy. According to the WHO’s recommendation [6], BEP supplements can be administered in settings/populations with more than 20% of underweight women of reproductive age (not in individual pregnant women identified as underweight).

### Appendix A.3. Background Information and Interview Questions

Please describe your current role and responsibilities related to health and nutrition in your country.

Topic 1: BEP supplements type, form, quantity

1. What is your experience with food supplementation, including balanced energy and protein BEP supplementation during pregnancy?
2. What do you know about BEP supplement interventions in pregnant and lactating women?
  a. Probe on familiarity with WHO recommendation or country guidelines on BEP supplementation.
3. What type of BEP is provided (ready-to-use products (e.g., lipid nutrient supplements, biscuits), fortified cereals or flours (e.g., corn/soya blend/CSB+), locally prepared foods and snacks?
4. What is the nutritional composition of the BEP supplement provided?
5. What is the target nutritional composition of BEP locally prepared foods and snacks?
6. What instructions are given on how to consume the product?
7. How much BEP supplement (e.g., quantity in serving, number of servings/doses) is given during pregnancy?
8. How was the BEP supplement type (e.g., product vs food) determined?
9. Which form of BEP supplement was the most acceptable in your setting?

Topic 2: Targeting strategies used

1. Who is receiving the BEP supplementation?
2. What criterion is used to determine who should receive BEP supplement?
3. How do you screen pregnant women to receive BEP supplement?
4. When is the BEP supplement delivered (e.g., pre-pregnancy/pregnancy/lactation-postpartum)?
5. In which trimesters do pregnant women typically receive the first BEP supplement package?
6. How long is the BEP supplement given for?
7. What are the criteria for exiting BEP supplementation?
8. Which potentially eligible pregnant women are/may not be reached using current platforms?
9. Are women who do NOT receive BEP supplement given any alternative information or service?
10. Where is the BEP supplement provided to recipients (during ANC or other platforms) within communities?
11. Who delivers the intervention to women?
12. What barriers/challenges do you face when prescribing BEP supplementation?

Topic 3: Cost & supply

1. What is the cost of the BEP supplement provided?
2. Who pays for BEP/food supplementation?
3. Who supplies the BEP/food supplementation in the country?
4. Where is BEP supplement stored before the delivery to points of distribution?

Topic 4: Point-of-distribution delivery and storage

1. Where do distribution sites obtain their BEP supplement?
2. Who delivers the BEP supplement to your area/facility?
3. Where is the BEP supplement stored before collection by recipients?
4. If any, what challenges are encountered with storage?
5. How were delivery locations/regions chosen?
6. If any, what criteria were used to choose the locations/regions?
7. Describe the availability of BEP/food supplementation at your facility/department.
8. What barriers/challenges do you face with BEP supplement deliveries?

Topic 5: Nutrition counseling

1. Where could women learn more about BEP supplements or food/snacks that qualify as a BEP supplement (where could they find this information)?
2. Are you aware of nutrition counseling services available for pregnant women or those planning to get pregnant?
3. If yes, who provides this type of information for pregnant women?
4. How much time do health practitioners spend with a woman to deliver nutrition counseling?
5. If not, what could be a feasible solution?

Topic 6: Advocacy and community/government readiness

1. What are your thoughts on community leaders and family members’ perception of BEP supplements?
2. What would enable community leaders and family members to advocate for a BEP supplement in undernourished women?
  a. Probe: Which community leaders and family members?
3. What would prevent community leaders or family members from advocating BEP supplement for undernourished women?
4. What type of resources do communities/local governments have to implement BEP supplement in undernourished women?
5. Which other organizations are willing to help undernourished women with a BEP supplement?

Topic 7: Monitoring process

1. How is BEP supplement administration monitored, including misuse (e.g., selling, food sharing, and intake monitoring)?
2. What type of implementation outcomes of BEP supplement are being monitored?
3. How often are these implementation outcomes monitored?
4. How would someone go about assessing if implementing BEP supplement works?
5. What strategies can promote acceptance/adherence to BEP supplementation?

Topic 8: Training materials for health staff in ANC setting or emergency/humanitarian setting (when interviewing Ob/Gyn or other health staff)

1. What helped you understand the importance of providing BEP supplementation to pregnant women?
2. What standard operating procedures or instructions are used for BEP supplement administration in women?
  a. Are you willing to share the training materials?
3. We briefly discussed food sharing earlier, how common is BEP supplement ‘food sharing’?
4. What type of guidance is being given to women to avoid BEP supplement ‘food sharing’?
5. What are the health effects you seek to achieve by providing the delivery of the BEP supplement?
6. How do you encourage women to take the BEP supplement?
7. What strategies have been implemented to raise awareness of BEP supplement interventions?
8. What strategies have been used to improve the acceptance of BEP supplement interventions?
9. What do women think of BEP supplement interventions?

Topic 9: For government-sponsored countries only

1. Does the country’s government fund BEP supplementation?
  a. If the country’s government funds BEP supplementation:
2. What process was undertaken for the local government to adopt BEP supplementation?
3. How was the local government convinced to adopt BEP supplementation?
4. How long has this intervention been in place?
5. If the intervention is no longer active, why did it stop?
6. What criteria is the government using to allocate BEP supplementation?
7. How many regions across the country are given BEP supplement interventions?
8. If only some, what obstacles prevent scaling up the intervention in other regions in need?
9. What type/form of BEP supplement intervention is given across different regions?
10. How is the government monitoring the success of the program?
11. Is the BEP supplement offered through the ANC?
  a. Are there enough staff to cover all necessary interventions for the needy population?
  b. If not, what solutions could be implemented to provide the BEP supplement through the ANC?
12. Is BEP offered through a different platform?
  a. What is the type of platform?
  b. Who manages it?
13. Are you <the government> willing to share monitoring data with us?
  a. Do you have additional thoughts/suggestions?
